# The association between chronotype and social anxiety among Chinese university students: a moderated mediation analysis of loneliness and perceived social support

**DOI:** 10.1186/s12889-024-20811-3

**Published:** 2024-11-29

**Authors:** Yingying Zhu, Junling Liu, Fulin Chen, Qian Wang, Kunxia Cao, Jiahao Huang, He Wang, Qiang Wang, Xue Luo

**Affiliations:** 1 Key Research Base of Humanities and Social Sciences of the, Ministry of Education, Academy of Psychology and Behavior, Tianjin Normal University, Tianjin, 300387 China; 2 Faculty of Psychology, Tianjin Normal University, Tianjin, 300387 China; 3 Tianjin Social Science Laboratory of Students’ Mental Development and Learning, Tianjin, 300387 China; 4 Institute of Biomedical Engineering, Chinese Academy of Medical Science & Peking Union Medical College, Tianjin, 300192 China; 5 Department of Psychiatry, Nanfang Hospital, Southern Medical University, Guangzhou, 510515 China

**Keywords:** Chronotype, Social anxiety, Loneliness, Perceived social support, Moderate mediation effect

## Abstract

**Background:**

Social anxiety has been a burning problem among contemporary college students in China. Increasing evidence suggests that individual circadian typology–chronotype may play an important role in the development of social anxiety. However, little research has focused directly on examining the association between chronotype and social anxiety, and less is known about the mediating and moderating mechanisms underlying this relationship. The aim of the present study was to investigate the link between chronotype and social anxiety among Chinese college students, and to explore the mediating effect of loneliness and the moderating effect of perceived social support in the association between chronotype and social anxiety.

**Methods:**

A cross-sectional design was conducted among 1616 college students (16–29 years old) from several public universities in Northern China, including 1172 females (72.52%) and 444 males (27.48%), with an average age of 19.68 years old (SD = 1.49). All participants completed the standardized self-report questionnaires including the Social Avoidance and Distress Scale, Morningness-Eveningness Questionnaire, UCLA Loneliness Scale, and the Perceived Social Support Scale. Common method bias was performed using Harman’s single-factor test. The mediation and moderation effects were analyzed using SPSS software and PROCESS macros.

**Results:**

Chronotype had a negative predictive effect on social anxiety in college students. Specifically, the greater the inclination of individuals' chronotypes toward evening preference, the more pronounced their symptoms of social anxiety would be. Loneliness served as a partial mediator in the relationship between chronotype and social anxiety, accounting for 30.0% of the total effect. In addition, perceived social support, particularly from friends and significant others, was found to play a moderating role in the process of loneliness affecting social anxiety among college students, while support from family did not. Interestingly, the perceived social support displayed a limited protective effect when college students suffered from higher levels of loneliness.

**Conclusion:**

These findings deepened our understanding of how and when (or for “whom”) chronotype is related to social anxiety, offering a theoretical foundation and practical insights for preventing and addressing social anxiety risk in young adult university students, particularly those with evening chronotypes.

## Introduction

Social anxiety is defined as an irrational persistent fear or anxiety of social situations that involve social interaction or social performance where the individual may be scrutinized, observed, or negatively evaluated by others [1]. Its clinical form is social anxiety disorder (SAD), also known as social phobia, which can lead to mental, physical, or social dysfunctions [2]. Social anxiety is becoming increasingly common among young adults, especially university students, due to rapid social development, academic demands, and increased pressures in the workforce. It has been estimated that approximately 16% of Chinese college students suffer from severe social anxiety [3], and 33.38% of undergraduate students reported that they have experienced at least one symptom of social anxiety [4]. Additionally, the lifetime prevalence of social anxiety among university students has exceeded 12% and is expected to continue to increase in the future [5]. As a maladaptive psychological problem, social anxiety can lead to a series of negative impacts on college students’ academic and personal lives. For example, socially anxious college students are prone to suffer from more academic maladjustment [6], experience higher levels of academic burnout [7], and achieve fewer educational milestones [8]. Besides, the higher the level of social anxiety among college students, the lower their self-esteem and individual subjective well-being would be [9, 10]. Recent empirical studies have even found that severe social anxiety is an enhancing-risk factor for triggering undergraduates’ suicide ideation and non-suicidal self-injury (NSSI) behaviors [11, 12]. Therefore, given the harmfulness of social anxiety on university students’ mental health and development, it is imperative to explore the contributing factors and the potential mechanisms through which social anxiety occurs, so as to formulate timely interventions and strategies for college students with social anxiety.

### Chronotype and social anxiety

Chronotype, also known as morningness-eveningness, is characterized by the differences in rest/activity or circadian timing between individuals [13]. It is a crucial indicator for evaluating individual circadian rhythm disturbances [14] and has been increasingly recognized as an important factor related to mental health [15]. Generally, chronotype can be divided into three types: morning- (MT), evening- (ET), and intermediate-type (IT) [16]. MT persons (so-called “larks”) prefer going to bed and waking up early, and experiencing peak performance and alertness around the morning hours. By contrast, ET persons (so-called “owls”) favour going to bed and waking up later, and prefer organizing activities towards the end of the day and evening hours [17]. The two-process sleep regulation theory has proposed that an individual’s sleep–wake (rest-activity) cycle is influenced by the interaction of the homeostatic sleep process and the circadian process, with the circadian process playing a particularly significant role. Disruption of the circadian rhythm (i.e., late chronotype) may lead to a range of health-related outcomes [18]. Many empirical studies have verified this theory by indicating that individuals with an evening preference are more prone to experience poorer sleep quality [19], increased daytime fatigue and sleepiness [20], severe substance abuse [21], as well as higher physical and verbal aggression behaviors [22]. Recently, there is evidence suggesting that eveningness may also be a risk factor for individual social anxiety. For instance, one cross-sectional study conducted by Azad-Marzabadi and Amiri [23] found that morningness-eveningness was associated with social anxiety, and can negatively predict social anxiety in undergraduate students. In other words, those with eveningness tend to exhibit higher levels of social anxiety compared to those with morningness. Another study by Markarian et al. [24] observed that individuals with an evening preference were more inclined to use experiential avoidance strategies to evade social activities. However, this avoidance as a maladaptive coping mechanism only provides temporary relief, which may consequently reinforce an individual’s fear of social situations, ultimately leading to a vicious cycle of social anxiety. Moreover, findings from some experimental studies have also shown that eveningness is associated with greater difficulties in managing social expectations and lower emotional regulation abilities [25, 26]. These factors have been proven to significantly contribute to social anxiety [27]. Despite empirical evidence supporting the connection between chronotype and social anxiety, the internal mechanisms of how and when or for whom chronotype is related to social anxiety remain largely unknown. To fill the research gap, the present study attempted to construct a moderated mediation model to explore the mediating role of loneliness and the moderating role of perceived social support among young college students.

### The potential mediating role of loneliness

Loneliness is defined as the aversive state experienced when a discrepancy exists between an individual’s wished and actual social relationships [28]. A growing number of studies indicate that loneliness is a significant predictor of social anxiety [29, 30]. The greater an individual’s loneliness, the higher the level of his or her social anxiety symptoms will be. Evidence from clinical research has found that after an eight-week cognitive-behavioral therapy treatment for lonely patients, their symptoms of social anxiety significantly decreased compared with the control group. This suggests loneliness could be a focal point for individual social anxiety prevention and interventions [31]. In addition, the loneliness model posits that feelings of loneliness can lead individuals to develop negative cognitive biases towards their surroundings and harbor pessimistic expectations for social interactions [32]. This, in turn, may result in severe social avoidance and anxiety symptoms [33]. Cross-lagged longitudinal studies have also concluded that pretest loneliness could significantly predict future occurrences of social anxiety [34].

On the other hand, chronotype, especially evening chronotypes, may be a susceptibility factor for loneliness. Based on the social jetlag theory [35], individuals with a preference for eveningness tend to experience a greater misalignment between their preferred sleep–wake times and conventional social schedules. This misalignment can result in them spending less time in the company of others. The reduced social contact or interaction may, in turn, precipitate a certain level of social isolation and subsequent loneliness [36, 37]. Investigations from recent cross-sectional and fMRI studies have also confirmed that eveningness is significantly associated with increased odds for reporting self-perceived loneliness, even after controlling for age, gender and sleep quality, whereas with no evidence for a similar association in neither-types [38]. Therefore, based on the theoretical and empirical grounds, it is reasonable for us to propose that loneliness would mediate the association between chronotype and social anxiety in college students.

### The potential moderating role of perceived social support

Social support is the practical, informational, and emotional assistance provided by a social circle that includes family, friends, and important individuals like peers, neighbors, and counselors [39]. It can be categorized into two types: received social support and perceived social support [40]. Perceived social support is broadly characterized by how individuals perceive the presence of social support in their networks, focusing on their subjective emotional experience and contentment when they feel valued, supported, and understood in society [41]. Compared with received social support, perceived social support has a stronger relationship with a person’s mental health [42]. As emphasized by the main effects model of social support [43], an individual’s positive perceptions of their social networks can help foster greater self-efficacy in managing adverse events, sustain a positive emotional state, enhance confidence, and reduce the likelihood of psychological and behavioral issues. Therefore, perceived social support may serve as a crucial positive psychological resource to alleviate the adverse impact of negative experiences or stressful events on individual physical and mental health. Prior studies have shown that perceived social support has a significant positive effect on reducing loneliness [44] and social anxiety [45]. A cross-sectional study by Xiao et al. [46] observed that perceived social support could effectively moderate the relationship between chronic diseases and loneliness in older adults, and the higher the perceived social support, the less chronic diseases affected loneliness. Among children and adolescents, researchers have found that those perceiving greater social support are more likely to engage in social activities, exhibit more prosocial behavior, and report fewer feelings of loneliness and social anxiety symptoms [47, 48]. Additionally, high levels of perceived social support can help decrease negative social and cognitive biases associated with loneliness [49], which, in turn, may contribute to diminishing negative emotions and behaviors associated with depression and social anxiety [29, 50]. Therefore, in line with the main effects model of social support and empirical research backgrounds, we assumed that perceived social support would play a moderating role in the process of loneliness affecting social anxiety among college students. Moreover, to the best of our knowledge, very few studies have been conducted to probe the beneficial effects of specific social support from various sources (i.e., family support, friends support, and significant others support) in the development of college students’ sleep and mental health. Accordingly, the current study also delved into the potential moderating effects of differentiated sources of social support in the influence of chronotype on social anxiety among college students.

### The present study

Although several studies have explored the link between chronotype and individuals’ social anxiety, the internal psychological mechanisms underlying the pathways of mental health impact remain unclear. Moreover, there have been a limited number of studies that have simultaneously examined the mediating and moderating mechanisms underlying the relationship between chronotype and social anxiety in young adults. Therefore, based on previous findings and relevant theories, the present study aimed to establish a moderated mediation model with loneliness as a mediating variable and perceived social support as a moderating variable to comprehensively investigate their roles in the process of chronotype affecting social anxiety among young college students. Specifically, our study would test the following hypotheses: (a). Chronotype may be negatively associated with social anxiety among college students (H1); (b). Loneliness may play a mediating role in the association between chronotype and social anxiety (H2); (c). Perceived social support, along with its sub-dimensions, may exert a moderating effect on the path of loneliness affecting social anxiety in college students (H3). This integrated model could, on one hand, address questions about both mediation (i.e., how individual chronotype preference associates with social anxiety) and moderation (i.e., for whom the relation is most or least strong). On the other hand, it has practical implications for offering interventions to protect college students from severe social anxiety, particularly those with late chronotypes.

## Methods

### Participants

This was a cross-sectional survey and participants came from several public universities in Tianjin and Hebei Province by convenient sampling method. All participants were invited to complete an online self-reported questionnaire that included demographic information, chronotype, loneliness, social anxiety, and perceived social support. They were informed in detail of the purpose and procedures of the study and provided informed consent before enrollment. After completing the survey, participants were given small gifts for their participation. A total of 1636 participants completed the questionnaire, and 20 of them were excluded due to missing values or incomplete questionnaires. Ultimately, 1616 students (72.52% females, 27.48%males; Mean age 19.68 ± 1.49 years) were included in the current analysis. The effective response rate was 98.7%. This study was reviewed and approved by the Research Ethics Committee of Tianjin Normal University (LL2023121302) and was thus performed in accordance with ethical standards laid down in the 1964 Declaration of Helsinki and its later amendments.

### Measurements

#### Predictor variable: chronotype

Chronotype was measured by the Morningness-Eveningness Questionnaire (MEQ) [51]. The Chinese version of MEQ has been proven to have good reliability and validity among university students [52]. This questionnaire consists of 19 items concerning individual’s preferred timing for sleep–wake cycles, physical and mental activity as well as subjective alertness. All items have a response scale with four or five options (items 3–9, 11–16 and 19 have four options, and items 1, 2, 10, 17, and 18 have five options). Sample items are “At what time would you get up if you were entirely free to plan your day?” and “During the first half-hour after having woken in the morning, how tired do you feel?”. Item scores were summed up to obtain a total score ranging from 16 to 86, with lower scores (≤ 41) indicating an evening preference, higher scores (≥ 59) indicating a morning preference, and intermediate falling between 42 and 58. In this study, the Cronbach’s α coefficient was 0.73.

#### Outcome variable: social anxiety

The Social Avoidance and Distress Scale (SADS) developed by Watson and Friend [53] was used to assess participants’ social anxiety. The SADS has been shown in Chinese samples with well-established validity and reliability [54]. The scale consists of 28 true/false items and is divided into two dimensions: social avoidance and social distress. Each item on the SADS is a statement concerning aspects of social anxiety. Sample items are “I am usually nervous with people unless I know them all” and “I often want to get away from people”. The total score is calculated by adding up all the scores on each item and ranges from 0 to 28. Higher scores represent higher levels of social anxiety. In this study, the Cronbach’s α coefficient was 0.88.

#### Mediator variable: loneliness

Participants’ loneliness was measured using the University of California at Los Angeles (UCLA) Loneliness Scale Short-form (ULS) [55], which consists of 8 items (six items worded in a negative/lonely direction and two items worded in a positive/non-lonely direction). Each item is rated on a four-point Likert scale ranging from 1 (“never”) to 4 (“often”). Sample items are “I lack companionship” and “There is no one I can turn to”. A composite score is computed by summing the items after reverse coding when appropriate. The total score ranges from 8 to 32, with a higher score indicating a greater level of loneliness. The Chinese version of ULS-8 has demonstrated satisfactory construct validity and internal reliability in undergraduate students [56]. The Cronbach’s α coefficient in this study was 0.81.

#### Moderator variable: perceived social support

The Multidimensional Scale of Perceived Social Support (MSPSS) compiled by Zimet et al. [57] was used to assess participants’ levels of perceived social support. The Chinese version was revised by Jiang [58]. This scale consists of three dimensions: family (e.g., “My family really tries to help me”), friends (e.g., “I can talk about my problems with my friends”), and significant other subscale (e.g., “There is a special person in my life who cares about my feelings”). Each dimension contains 4 items and participants were asked to rate these items on a seven-point Likert scale ranging from 1 (“very strongly disagree”) to 7 (“very strongly agree”). The level of perceived social support is reflected by a total score (range from 12 to 84), higher scores represent a higher level of individuals’ perceived social support. In this study, the Cronbach’s α coefficient for the total scale was 0.96, and the Cronbach’s α for the family, friends, and significant other subscales were 0.91, 0.93, and 0.91, respectively.

#### Covariates

In addition to the substantive variables of interest, we controlled for some sociodemographic characteristics of the participants in this study, including age (in years), gender (male versus female), academic major (humanities, science, or other fields), grade level (freshman, sophomore, junior, senior, or postgraduate), and family type (whether the participant came from a single-child family or not, categorized as only child versus non-only child).

### Statistical analysis

Statistical analyses were processed using SPSS 26.0 (IBM) and the PROCESS 4.0 macro program. First, descriptive statistics for all study variables and Person’s correlation for the key variables were generated. Frequencies and proportions were used to describe categorical variables, and continuous data were represented as mean ± standard deviation *(M* ± *SD).* Second, SPSS PROCESS 4.0 macro was used for mediation and moderated mediation analyses, with bootstrapping set to 5000. Specifically, the simple mediation effect of loneliness on the association between chronotype and social anxiety was tested using Model 4 in PROCESS macro version 4.0. The indirect effect of chronotype on social anxiety through loneliness was defined as the product of a (the effect of chronotype on loneliness) and b (the effect of loneliness on social anxiety). Model 14 in PROCESS macro version 4.0 was performed to test whether perceived social support played a moderating effect on the second half of the mediation model above. A simple slope analysis was examined at one standard deviation above and below the mean of perceived social support if the interaction of moderation effect was significant. Third, to further explore the moderation effects of perceived social support from different sources, family, friends, and significant other support were also tested as moderators by Model 14, with the *post-hoc* simple effects analysis for significant moderators. All continuous study variables were standardized. The indirect effect in the simple mediation model and conditional indirect effect in the moderated mediation model were calculated using 5000 bootstrap samples with 95% bias-corrected confidence intervals (CIs), whereby a non-zero bootstrap confidence interval indicates a significant effect [59]. Additionally, prior research has shown that college students’ loneliness and social anxiety can vary based on demographic factors such as gender, age, education level, and family type or structure [60, 61]. Building on these previous findings, we included several sociodemographic variables that might potentially influence the dependent variable as covariates in the statistical models to enhance the reliability of the study. Moreover, *P* values < 0.05 were considered statistically significant and should be interpreted in the context of the confidence intervals.

## Results

### Preliminary analysis

Harman’s single-factor method was first applied to test common method bias and systematic errors due to self-report questionnaires. Principal component analysis of all variables extracted eleven eigenvalues greater than 1. The first factor accounted for 15.86% of the total variation, which was less than the critical standard of 40% [62]. It suggested that there was no serious problem of common method bias in the present study.

### Descriptive statistics

The sociodemographic characteristics of participants, along with the demographic variance analysis of social anxiety are presented in Table 1. A total of 1616 college students were included in the present study, which comprised 1172 females and 444 males. Among the participants, 324 came from only-child families and 1292 from non-only child families. There were, in order of enrollment, 1111 freshmen, 286 sophomores, 105 juniors, 58 seniors, and 56 postgraduates. Results of one-way analysis of variance showed that social anxiety displayed significant differences among age variables (*F*(2, 1613) = 3.30, *p* < 0.05). The social anxiety scores of students aged between 16 and 20 were significantly higher than those of students aged between 21 and 25 (*p* < 0.05). There were significant differences in social anxiety among different grades (*F*(4, 1611) = 10.71, *p* < 0.01). The social anxiety scores of freshmen and seniors were higher than that of sophomores (*p* < 0.05). College students majoring in the humanities had significantly higher social anxiety scores than those in the sciences (*p* < 0.05). In addition, there was also a significant difference in social anxiety among students with different chronotypes (*F*(2, 1613) = 25.37, *p* < 0.001). Compared with students with morning and intermediate chronotypes, evening-type students demonstrated higher social anxiety scores (*ps* < 0.01).

**Table 1:** Means (M), standard deviations (SD), and demographic variance analysis of social anxiety among participants (*N* = 1616)

Variables	*n (%)*	*M (SD)*	*t/F*
Age 16–20	1265 (78.28)	13.81 (6.75)	3.30^*^
21–25	344 (21.29)	12.80 (6.70)	
26–30	7 (0.43)	15.43 (6.21)	
Gender Male	444 (27.48)	13.22 (6.22)	1.50
Female	1172 (72.52)	13.75 (6.93)	
Grade Freshman	1111 (68.75)	14.20 (6.76)	10.71^**^
Sophomore	286 (17.70)	11.42 (6.40)	
Junior	105 (6.50)	12.68 (5.96)	
Senior	58 (3.59)	14.66 (7.42)	
Postgraduate	56 (3.47)	13.68 (6.54)	
Major Humanities	615 (38.06)	14.09 (6.73)	3.34^*^
Science	848 (52.48)	13.20 (6.68)	
Others	153 (9.47)	13.90 (7.09)	
Family type Only child	324 (20.05)	13.62 (6.59)	0.06
Non-only child	1292 (79.95)	13.60 (6.79)	
Chronotype Morning-type	222 (13.74)	11.90 (7.04)	25.37^***^
Intermediate-type	1143 (70.73)	13.48 (6.47)	
Evening-type	251 (15.53)	16.20 (7.09)	

^*^p < 0.05, ^**^p < 0.05, ^***^p < 0.001

Table 2 presents the bivariate correlations of the main study variables. The results showed that chronotype was significantly and negatively associated with social anxiety (*r* = − 0.20, *p* < 0.001) and loneliness (*r* = − 0.12, *p* < 0.001). Loneliness was significantly and positively correlated with social anxiety (*r* = 0.51, *p* < 0.001) as well. In addition, perceived social support and its three dimensions (family, friends, and significant other) were found to be significantly and negatively correlated with lower loneliness (*ps* < 0.001) and lower social anxiety (*ps* < 0.001), respectively.

**Table 2:** Mean, standard deviation and correlations between main study variables

Variables	M	SD	1	2	3	4	5	6	7
Chronotype	50.33	8.30	1						
Social anxiety	13.60	6.75	− 0.20^***^	1					
Loneliness	16.17	4.46	− 0.12^***^	0.51^***^	1				
Perceived social support	62.80	13.29	− 0.03	− 0.18^***^	− 0.32^***^	1			
Perceived family support	20.93	4.85	0.00	− 0.16^***^	− 0.27^***^	0.91^***^	1		
Perceived friends support	21.10	4.71	− 0.04	− 0.17^***^	− 0.32^***^	0.94^***^	0.76^***^	1	
Perceived significant other support	20.78	4.69	− 0.05	− 0.18^***^	− 0.30^***^	0.95^***^	0.78^***^	0.87^***^	1

^***^p < 0.001

### Testing for the mediation effect

**Fig. 1:**
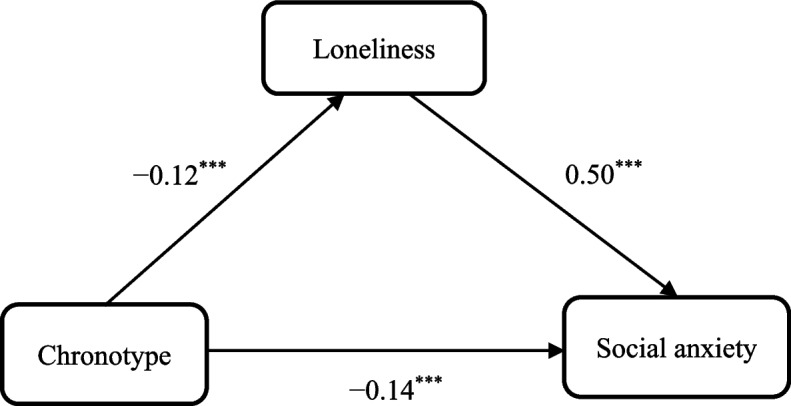
The mediating effect of loneliness on the relationship between chronotype and social anxiety..^***^p < 0.001

Hayes’s PROCESS (Model 4) was used to test the mediating role of loneliness in the association between chronotype and social anxiety. The mediation analysis results are shown in Table 3 and Fig. 1. Age, gender, grade, major, and family type were entered as covariates. Chronotype was recorded as the independent variable, social anxiety as the outcome variable, and loneliness as the mediating variable in the analysis. As shown in Table 3, when the mediating variable was not included, chronotype negatively predicted social anxiety (*β* = − 0.20, *t* = − 8.00, *p* < 0.001). After adding the mediating variable, chronotype significantly and negatively predicted loneliness (*β* = − 0.12, *t* = − 4.84, *p* < 0.001). Loneliness positively predicted social anxiety (*β* = 0.50, *t* = 23.26, *p* < 0.001). The direct effect of chronotype on social anxiety was also significant (*β* = − 0.14, *t* = − 6.39, *p* < 0.001). Moreover, the bootstrap 95% CI confirmed the significant indirect effect of loneliness in the relationship between chronotype and social anxiety (*β* = − 0.06, *SE* = 0.01, Boot 95% CI = [− 0.09, − 0.03]). These results indicated that loneliness had an indirect effect on the relationship between chronotype and social anxiety, that was, loneliness played a partial mediating role, and the mediating effect (*β* = − 0.06) accounted for 30.0% of the total effect (*β* = − 0.20). Thus, Hypotheses 1 and 2 were supported.

**Table 3:** The mediation effect of chronotype on social anxiety

Predictors	Model 1 (Social anxiety)			Model 2 (Loneliness)			Model 3 (Social anxiety)		
	*β*	*SE*	*t*	*β*	*SE*	*t*	*β*	*SE*	*t*
Chronotype	− 0.20	0.02	− 8.00^***^	− 0.12	0.03	− 4.84^***^	− 0.14	0.02	− 6.39^***^
Loneliness							0.50	0.02	23.26^***^
*R*^*2*^	0.04			0.02			0.28		
*F*	12.22^***^			5.88^***^			91.27^***^		

*SE* standard error. Age, gender, grade, major and family type were controlled as covariates in the analysis. ^***^p < 0.001

### Testing for the moderated mediation effect

The macro PROCESS (Model 14) was performed to test the moderated mediation model, in which chronotype was included as an independent variable, social anxiety as a dependent variable, loneliness as a mediator and perceived social support as a moderator. Gender, age, grade, major and family type were set as covariates. The results showed that chronotype was negatively associated with loneliness (*β* = − 0.12, *t* = − 4.84, Boot 95% CI = [− 0.17, − 0.07]) and social anxiety (*β* = − 0.14, *t* = − 6.47, Boot 95% CI = [− 0.18, − 0.10]). Loneliness was positively related to higher social anxiety (*β* = 0.49,* t* = 21.62, Boot 95% CI = [0.44, 0.53]). In addition, as shown in Table 4, the interaction term of loneliness × perceived social support was positively linked to social anxiety (*β* = 0.05, *t* = 2.36, Boot 95% CI = [0.01, 0.09]), and the 95% CI in conditional indirect effects showed both the low-level group and the high-level group does not contain zero, indicating that the effect of loneliness on social anxiety was moderated by the levels of perceived social support. Thus, Hypothesis 3 was supported.

**Table 4:** The moderated mediation effect of chronotype on social anxiety

Predictor variable	Model 1^a^			Model 2^b^			Model 3^c^			Model 4^d^		
	***β***	***t***	**95% CI**	***β***	***t***	**95% CI**	***β***	***t***	**95% CI**	***β***	***t***	**95% CI**
Chronotype	− 0.14	− 6.47^***^	[− 0.18, − 0.10]	− 0.14	− 6.43^***^	[− 0.18, − 0.10]	− 0.14	− 6.41^***^	[− 0.18, − 0.10]	− 0.14	− 6.54^***^	[− 0.18, − 0.10]
Loneliness	0.49	21.62^***^	[0.44, 0.53]	0.49	22.07^***^	[0.44, 0.53]	0.49	21.93^***^	[0.45, 0.54]	0.48	21.53^***^	[0.44, 0.52]
Perceived social support	− 0.04	− 1.54	[− 0.08, 0.01]									
PSS × Loneliness	0.05	2.36^*^	[0.01, 0.09]									
Family support				− 0.03	− 1.42	[− 0.08, 0.01]						
Family support × Loneliness				0.02	0.99	[− 0.02, 0.06]						
Friends support							− 0.02	− 0.78	[− 0.06, 0.03]			
Friends support × Loneliness							0.07	3.38^***^	[0.03, 0.11]			
Significant others support										− 0.06	− 2.44^*^	[−0.10, −0.01]
Significant others support × Loneliness										0.05	2.48^*^	[0.01, 0.09]
*R*^*2*^	0.29			0.28			0.29			0.29		
*F*	72.28^***^			71.38^***^			72.83^***^			72.91^***^		

*PSS* Perceived Social Support.^*^p < 0.05, ^***^p < 0.001

^a^Model 1 Chronotype as an independent variable, social anxiety as a dependent variable, loneliness as a mediator and moderated by PSS, adjusted for age, gender, grade, major and family type

^b^Model 2 Chronotype as an independent variable, social anxiety as a dependent variable, loneliness as a mediator and moderated by family support, adjusted for the covariates in Model 1

^c^Model 3 Chronotype as an independent variable, social anxiety as a dependent variable, loneliness as a mediator and moderated by friends support, adjusted for the covariates in Model 1

^d^Model 4 Chronotype as an independent variable, social anxiety as a dependent variable, loneliness as a mediator and moderated by significant others support, adjusted for the covariates in Model 1

**Fig. 2:**
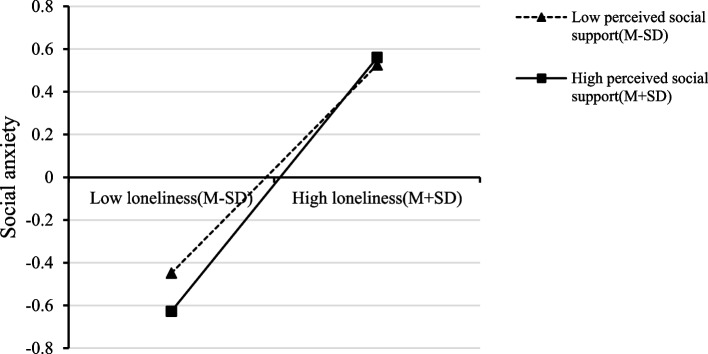
The moderating effect of perceived social support on the association between loneliness and social anxiety

**Fig. 3:**
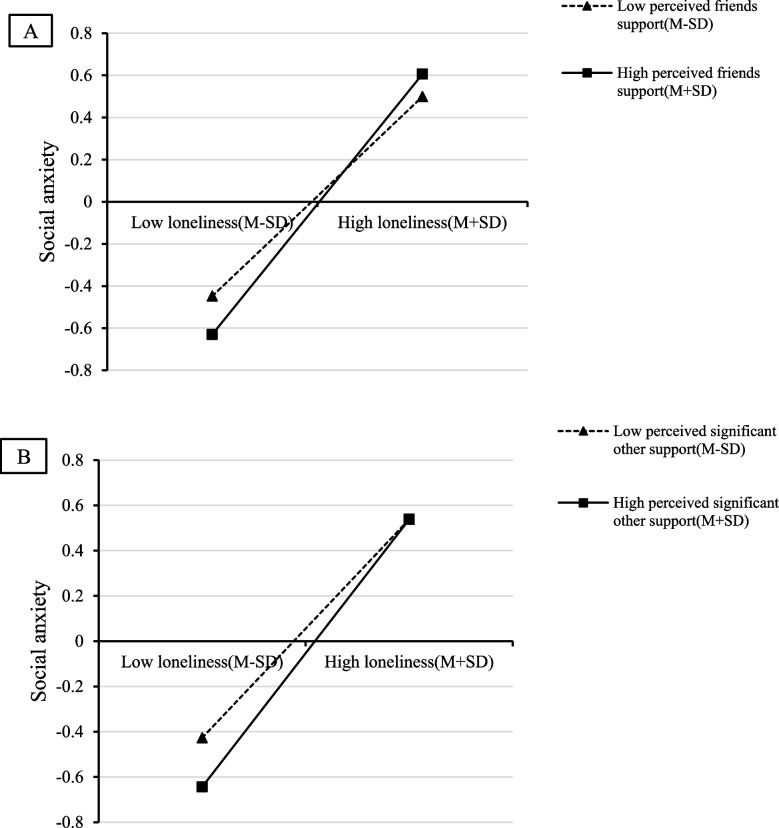
**A**. The moderating effect of perceived friends support on the association between loneliness and social anxiety; (**B**). The moderating effect of perceived significant others support on the association between loneliness and social anxiety

To better explain the moderating effect of perceived social support, participants were divided into a high perceived social support group (Mean + 1SD) and a low perceived social support group (Mean − 1SD) based on the average score of perceived social support plus or minus one standard deviation (see Fig. 2). The results showed that individuals with higher perceived social support reported lower levels of social anxiety than those with lower levels of perceived social support with the increasing loneliness. We subsequently tested the specific moderating effect of three sub-dimensions of perceived social support to examine the distinct influence of social support from various sources on college students. The results, as shown in Table 4, revealed that the effect of the loneliness-by-friends support interaction on social anxiety was significant (*β* = 0.07, *t* = 3.38, Boot 95% CI = [0.03, 0.11]), and so was that of the interaction of loneliness-by-significant other support on social anxiety (*β* = 0.05, *t* = 2.48, Boot 95% CI = [0.01, 0.09]). The effect of the interaction between perceived family support and loneliness on social anxiety was not significant (*β* = 0.02, *t* = 0.99, Boot 95% CI = [− 0.02, 0.06]). To clearly describe the specific interaction effect of perceived support from friends and significant others, we plotted loneliness on social anxiety separately at low and high levels of social support (1SD above the mean and 1SD below the mean, see Fig. 3). The results indicated that for individuals with a low level of loneliness, the levels of social anxiety in high-score groups of perceived support from friends (Fig. 3A) or significant others (Fig. 3B) were significantly lower than those in low-score groups. The findings suggest that college students would benefit more from the support of friends and significant others when experiencing lower levels of loneliness.

## Discussion

The present study proposed a moderated mediation model to examine the relationship between chronotype on social anxiety among college students, along with its underlying mechanisms. To our knowledge, this study is among the first to explore the mediating role of loneliness and the moderating role of perceived social support and its sub-dimensions between chronotype and social anxiety spontaneously. The obtained results showed that chronotype is significantly and negatively associated with social anxiety in college students, and loneliness plays as a “bridge” between chronotype and social anxiety. Perceived social support, particularly support from friends and significant others, has a certain moderating effect on the indirect path from chronotype to social anxiety. These findings extend the academic understanding on the inner mechanisms of how chronotype may affect social anxiety, and additionally could assist educators and psychological professionals in developing more targeted interventions and prevention to alleviate social anxiety among college students.

As expected, the overall model revealed a significant correlation between chronotype and social anxiety in Chinese college students, and chronotype negatively predicted social anxiety, even after controlling for demographic variables such as age, gender, grade, family structure, and study major. The results were consistent with previous studies indicating that chronotype is closely related to social anxiety. Individuals with an eveningness chronotype were more prone to engage in a higher level of social anxiety symptoms compared to those with morning and intermediate chronotypes [23]. Prior research has shown that circadian dysfunction due to a late chronotype can cause difficulties in emotion regulation [63]. The impaired capacity for emotional regulation hinders individuals from effectively managing their concerns about social situations, ultimately leading to social anxiety [64]. Besides, evening-type persons were found to show a higher tendency to use counterproductive emotion regulation strategies, such as experiential avoidance and suppression, to deal with negative emotions like social anxiety [24, 65]. However, these maladaptive and unhealthy strategies for emotion regulation may, in turn, exacerbate negative emotional experiences and contribute to more severe emotional problems in the long term [66]. Additionally, a recent study by El-Jaziz and Lotfi [67] also demonstrated an association between eveningness and lower levels of extraversion, which has been proven to be highly correlated with social anxiety among university students. These findings suggest that the inclination to be an evening type can be a risk factor for the development of social anxiety among college students, while the inclination to be a morningness may act as a protective factor against social anxiety. This might be helpful for university teachers and educators to take some interventions aimed at assisting college students in establishing healthy sleep hygiene practices and shielding them from the negative effects of social anxiety.

In addition, this study extends the prior literature by showing that loneliness partially mediated the relationship between chronotype and social anxiety, with a mediating effect size of 30.0%. In other words, chronotype can not only affect college students’ social anxiety directly, but also can affect social anxiety through the partial mediating role of loneliness. This mediation model was consistent with previous studies that have suggested a positive correlation between late chronotype and greater levels of loneliness [68]. Compared to morning and intermediate types, evening types were more susceptible to loneliness. The finding can be explained by the social jetlag theory raised by Wittmann et al. [35]. According to this theory, people who prefer staying up late often face a challenge in synchronizing their preferred sleep and wake times with typical social schedules. As a result, they may end up spending less time with others, leading to decreased social interaction. The reduced contact can then contribute to feelings of social isolation and loneliness. Some researchers have also found that eveningness has been associated with problematic mobile phone use [69], and those with evening chronotypes tend to perceive excessive mobile phone use as a dysfunctional emotional regulation strategy to cope with negative emotions such as loneliness. Nevertheless, this negative coping mechanism may actually cultivate more detrimental consequences, creating a feedback loop that exacerbates feelings of loneliness and other negative emotions [70, 71]. Additionally, higher levels of loneliness could lead to an increased risk of social anxiety. It has been shown that relative to non-lonely people, lonely individuals typically do not evaluate themselves more positively in social interactions and are inclined to believe that they are not liked, included, or accepted by others [72]. Due to the absence of interpersonal self-positivity bias, lonely individuals are less likely to engage in social activities, tend to perform poorly in social situations, and may struggle to form and sustain meaningful relationships with others. These factors can contribute to more severe symptoms of social anxiety among lonely individuals [73]. Findings from longitudinal studies have further demonstrated that high levels of loneliness exert a significant predictive effect on social anxiety [34]. Therefore, the mediating mechanism of loneliness suggests that psychological programs or strategies designed to reduce loneliness, particularly for those with evening preference are essential for lowering the risk of social anxiety among young adult college students.

The results of this study also confirmed that perceived social support played a moderating role in the association between chronotype and social anxiety. Specifically, perceived social support had a moderating effect on the indirect path of loneliness affecting social anxiety, which supports our Hypothesis 3. Many previous studies have demonstrated the beneficial effects of social support on both mental and physical health [74–79]. On one hand, it has been shown that people who perceive a higher degree of social support tend to have a more optimistic mindset and greater satisfaction with their social relationships, which may make them less susceptible to emotional problems, such as depression and social anxiety [75]. For children and adolescents with anxiety disorders, social support played an important role in alleviating their anxiety symptoms [76]. On the other hand, social support can promote health maintenance behaviors by boosting self-esteem, enhancing a sense of control over health conditions, and increasing confidence in self-care abilities in heart failure patients [77]. Additionally, social support may improve treatment adherence in diabetes patients by reducing feelings of abandonment, rejection, and loneliness, while fostering greater trust in others [78] (see [79] for further details on the impact of social support on other physical illnesses). In line with prior research, we observed that the social anxiety levels of college students with high perceived social support were significantly lower as compared with those with low perceived social support levels. Moreover, the protective effect of perceived social support appeared stronger when individuals were at lower levels of loneliness. This finding was consistent with the reverse stress-buffering model which suggests that the protective effect of social support can be limited [80]. When college students experience lower levels of loneliness, social support can protect them from being heavily affected by it. Higher levels of loneliness, however, might harm individuals’ sensitivity to social relationships, making them less likely to feel and receive support from their social networks. Thus, the limited positive effects of perceived social support will be overshadowed by the detrimental effects of higher levels of loneliness. Furthermore, the results of the present study revealed for the first time that social support from friends and significant others could significantly buffer the adverse association between loneliness and social anxiety among college students, whereas support from families did not have a moderating effect on the relationship between loneliness and social anxiety. The possible explanation may be that when individuals enter university life, the social environment in which they operate undergoes significant changes. Individuals start to move away from primary dependency on their families of origin for intimacy and companionship, and instead, they begin to turn to friends and certain significant others, such as romantic partners [81]. Peer support and assistance can help individuals better adapt to new environments, and gain a sense of belonging, and security [82, 83]. These findings added an important piece to the literature by suggesting that the protective effect of social support on individuals’ mental health also varies depending on its sources. For college students, support from friends and significant others, rather than family support, would serve as an important buffer against the adverse psychosocial effects of stress, such as loneliness and social anxiety.

Several limitations of this study should be acknowledged. First, all the data in this study were retrospective and self-reported, which may increase the likelihood of reporting bias. Future studies are warranted to apply multiple measurements (such as the combination of objective and subjective assessment tools) to collect data and reduce subjective influence. Second, the use of cross-sectional design did not allow the establishment of causal inferences regarding the study variables. Tracking research or longitudinal studies that explore the causal link between chronotype, loneliness, and social anxiety is essential for gaining a deeper understanding of the intricate interplay between these variables. Third, the current study primarily focused on the impact of circadian typology-chronotype on college students’ social anxiety and the underlying mechanisms involved. There may be other important factors that can influence the relationship between chronotype and social anxiety. In the future, researchers should conduct further investigations into the relationship between various factors and social anxiety to better comprehend its internal mechanisms and provide a scientific basis for more comprehensive mental health enhancement among university students. Finally, the sample were obtained from some public universities in China. Thus, generalizations of the findings to other regions and populations must be made with caution. More research is expected to replicate and expand upon the findings of this study by including a more diverse sample of individuals from different regions and occupations, utilizing mixed-method approaches.

## Conclusions

Overall, the results of the present study not only provide further support for the connection between chronotype and social anxiety but also elucidate the potential mechanisms underlying the link between chronotype and social anxiety among young adult university students. Notably, loneliness was found to play a partial mediating role in the relationship between chronotype and social anxiety. Perceived social support, especially support from friends and significant others, moderates the pathway through which loneliness influences social anxiety. The findings addressed the question of how chronotype affects the social anxiety in college students, highlighting the negative predictive impact of chronotype on social anxiety and identify conditions under which the mediating effect of loneliness and the moderating effect of perceived social support are more pronounced. It is important to note that implementing efficient programs and interventions to reduce loneliness among college students and enhance their social support can be beneficial in decreasing social anxiety, especially for those with evening chronotypes.

## Data Availability

The datasets used and/or analyzed during the current study are available from the corresponding author on reasonable request.
